# Primary renal diffuse large B cell lymphoma misdiagnosed as renal cell carcinoma: A case report

**DOI:** 10.1097/MD.0000000000051228

**Published:** 2026-10-23

**Authors:** Xintong Li, Jia Li, Shijie Sun, Pintong Huang

**Affiliations:** a Department of Ultrasound, The Second Affiliated Hospital School of Medicine, Zhejiang University, Hangzhou, Zhejiang Province, China.

**Keywords:** case report, contrast-enhanced ultrasonography, diffuse large B-cell lymphoma, primary renal lymphoma, ultrasound-guided renal needle biopsy

## Abstract

**Rationale:**

Primary renal lymphoma (PRL) is a rare malignant lymphoma characterized by an extranodal presentation with limited or absent involvement of lymphatic channels. Its nonspecific clinical manifestations often make differentiation from renal cell carcinoma (RCC) challenging. This report describes a case of non-Hodgkin lymphoma initially diagnosed as RCC and subsequently confirmed as primary diffuse large B-cell lymphoma (DLBCL).

**Patient concerns:**

A 78-year-old man presented with right flank pain radiating to the abdomen for 2 weeks. Physical examination revealed no significant abnormalities.

**Diagnoses:**

Laboratory tests showed a mild elevation of lactate dehydrogenase (LDH) and positive urine occult blood. Imaging evaluations, including ultrasonography, computed tomography (CT), and magnetic resonance imaging (MRI), were suggestive of RCC. Contrast-enhanced ultrasonography further supported this initial impression.

**Interventions:**

Based on the suspicion of RCC, an ultrasound-guided renal needle biopsy was performed to establish the diagnosis.

**Outcomes:**

Histological Pathology: Histopathological analysis of the ultrasound-guided biopsy confirmed primary renal DLBCL.

**Lessons:**

This case underscores the importance of accurate diagnostic methods in oncology, particularly in distinguishing PRL from RCC, which can be challenging based on imaging alone. The findings demonstrate the utility of ultrasound-guided biopsy as a reliable diagnostic tool for primary renal lymphoma.

## 1. Introduction

Primary renal lymphoma (PRL) is defined as a non-Hodgkin lymphoma (NHL) involving the kidney in the absence of primary extrarenal lymphatic disease.^[1]^ Its existence was long debated because the kidney is an extranodal organ devoid of lymphatic tissue.^[1]^ PRL is extremely rare, accounting for approximately 0.7% of extranodal lymphomas in North America and 0.1% of all malignant lymphomas in Japan.^[2,3]^ It is predominantly a B-cell lymphoma, with diffuse large B-cell lymphoma (DLBCL) being the most common subtype.^[4]^ Because the clinical and imaging features of PRL are nonspecific and frequently overlap with those of renal cell carcinoma (RCC), preoperative differentiation between PRL and RCC is challenging, and renal biopsy is essential for establishing the diagnosis. We report the case of a 78-year-old man with flank pain who was initially diagnosed with RCC but was later confirmed to have primary DLBCL following an ultrasound-guided renal needle biopsy. Renal biopsy is a key modality for confirming PRL, providing pathological and immunohistochemical evidence that can prevent unnecessary surgery due to misdiagnosis and guide subsequent chemotherapy.

## 2. Case presentation

A 78-year-old man presented to our hospital with right flank pain radiating to the abdomen for 2 weeks. He reported no history of hematuria, fever, weight loss, night sweats, persistent fatigue, appetite loss, cough, chest pain, or abdominal pain. There was no family history of malignancies or genetic diseases. Physical examination revealed no abnormalities. Routine blood tests were within normal limits. Additional laboratory results were as follows: creatinine 136.3 µmol/L (normal range 57.0–115.0), urea 9.35 mmol/L (normal range 2.80–7.60), estimated glomerular filtration rate 42.5 mL/min (normal ≥ 90), and slightly elevated lactate dehydrogenase (LDH) 268 U/L (normal < 248). Urinalysis showed occult blood (2+). HIV serology was negative.

Abdominal ultrasonography (Fig. 1) showed a mildly enlarged mass, measuring approximately 7.28 × 6.71 cm, in the right kidney, with heterogeneous hypoechoic invasion of the right liver (Segment VI). The mass presented with an irregular shape, and color Doppler imaging detected internal blood flow signals. Additionally, a 3.89 × 3.11 cm hypoechoic mass with internal blood flow signals was observed in the para-aortic region. On non-contrast CT, an 83 × 53 mm mass lesion was identified at the right renal hilum, involving the upper-middle pole of the right kidney and partially involving the renal pelvis and calyces. The lesion appeared as a slightly hypodense heterogeneous mass with a mean attenuation of 39 HU. After contrast administration (Fig. 2), the mass exhibited mild to moderate heterogeneous enhancement, with mean attenuation values of 60 HU in the corticomedullary phase, 97 HU in the nephrographic phase, and 94 HU in the excretory phase. Tumor thrombus extending from the right renal vein to the L1–L2 intervertebral space was detected. An enlarged retroperitoneal lymph node at the L1 level showed prominent enhancement, with mean attenuation values of 57 HU in the arterial phase, 98 HU in the portal venous phase, and 109 HU in the delayed phase. Contrast-enhanced magnetic resonance imaging (CE-MRI) (Fig. 3) revealed that the renal mass demonstrated low signal intensity on T1-weighted imaging (T1WI) and high signal intensity on T2-weighted imaging (T2WI). The lesion showed high signal intensity on diffusion-weighted imaging (DWI) and significantly decreased signal intensity on the apparent diffusion coefficient (ADC) map, consistent with restricted diffusion. Homogeneous moderate enhancement of the mass was observed on CE-MRI. Based on comprehensive imaging findings – including a heterogeneous solid component, invasive involvement of the renal pelvis and calyces, renal vein tumor thrombus, and enlarged, enhancing retroperitoneal lymph nodes – the lesion was initially suspected to be RCC.

**Figure 1.**
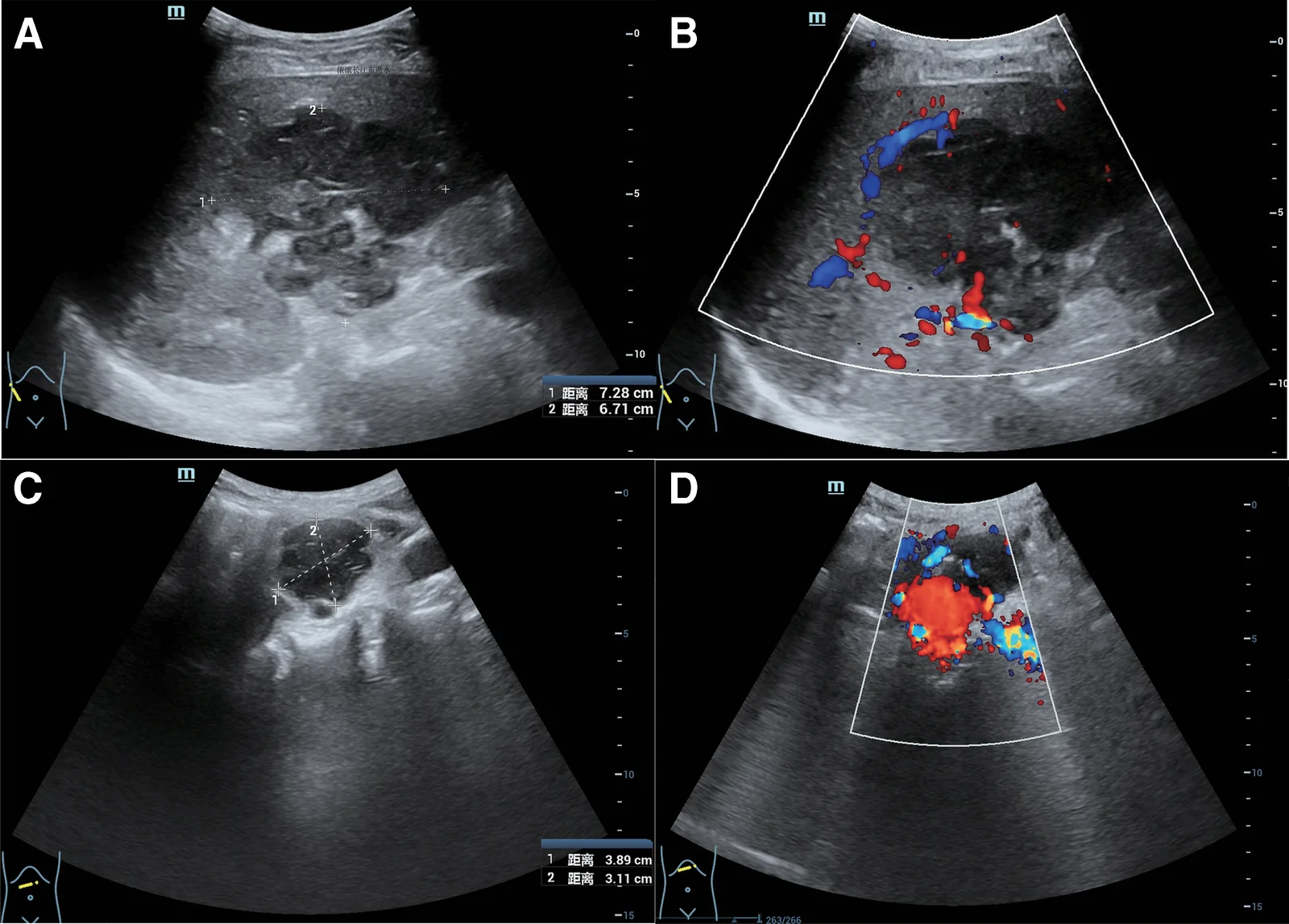
B-mode ultrasound. (A) A large right renal mass measuring 7.28 × 6.71 cm; the mass is hypoechoic with involvement of the right liver. (B) Color Doppler image showing intralesional blood flow signals. (C) A 3.89 × 3.11 cm hypoechoic lesion in the para-aortic region. (D) Color Doppler image showing blood flow signals within the para-aortic lesion.

**Figure 2.**
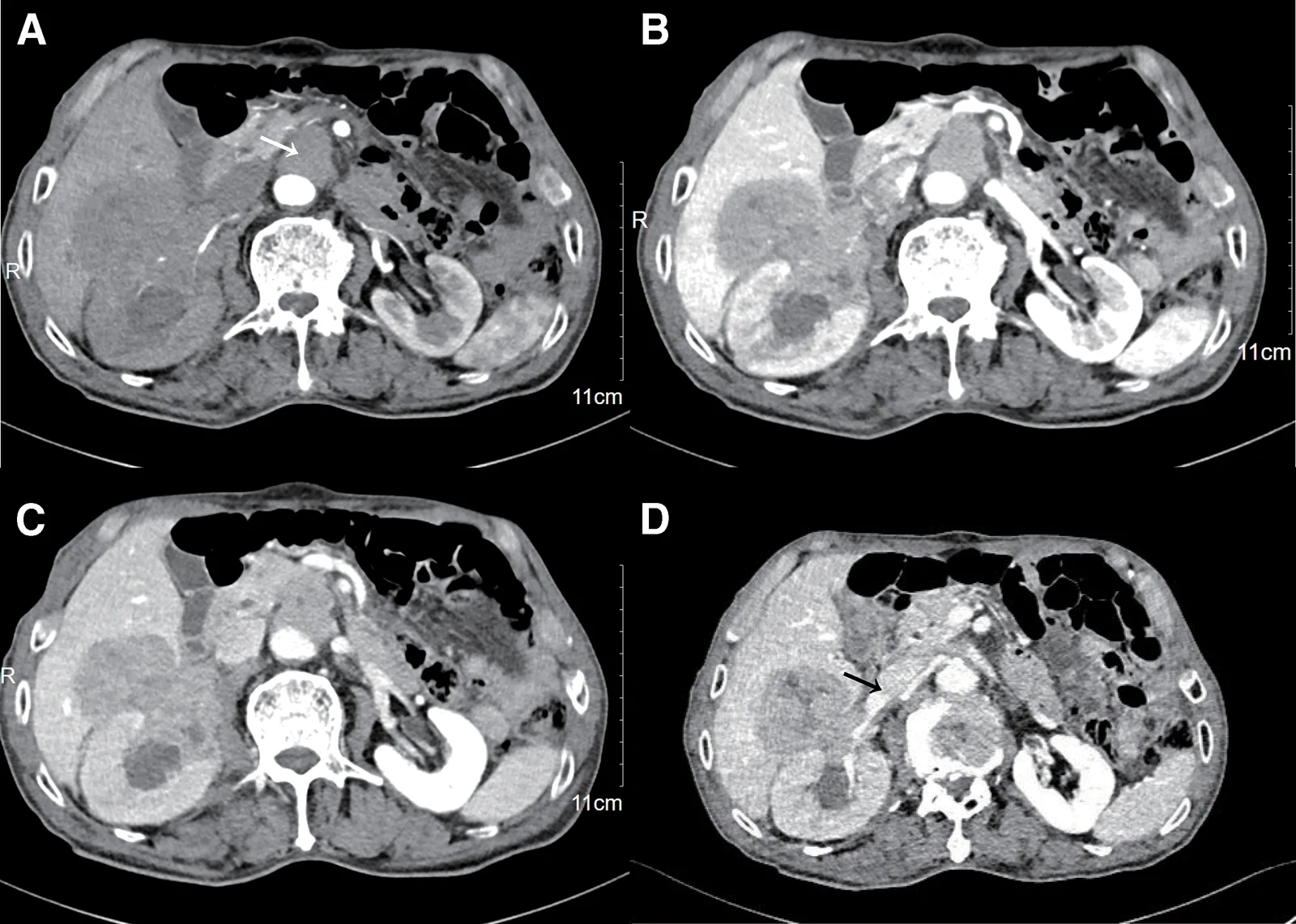
Contrast-enhanced CT (CE-CT). Corticomedullary phase (A), Nephrographic phase (B), and Excretory period (C). A CE-CT scan of the right kidney reveals a slightly hypodense, heterogeneous mass measuring 83 × 53 mm with mild to moderate enhancement that infiltrates the renal vessels (D, black arrow), along with a para-aortic lymph node measuring 34 × 31 mm (A, white arrow).

**Figure 3.**
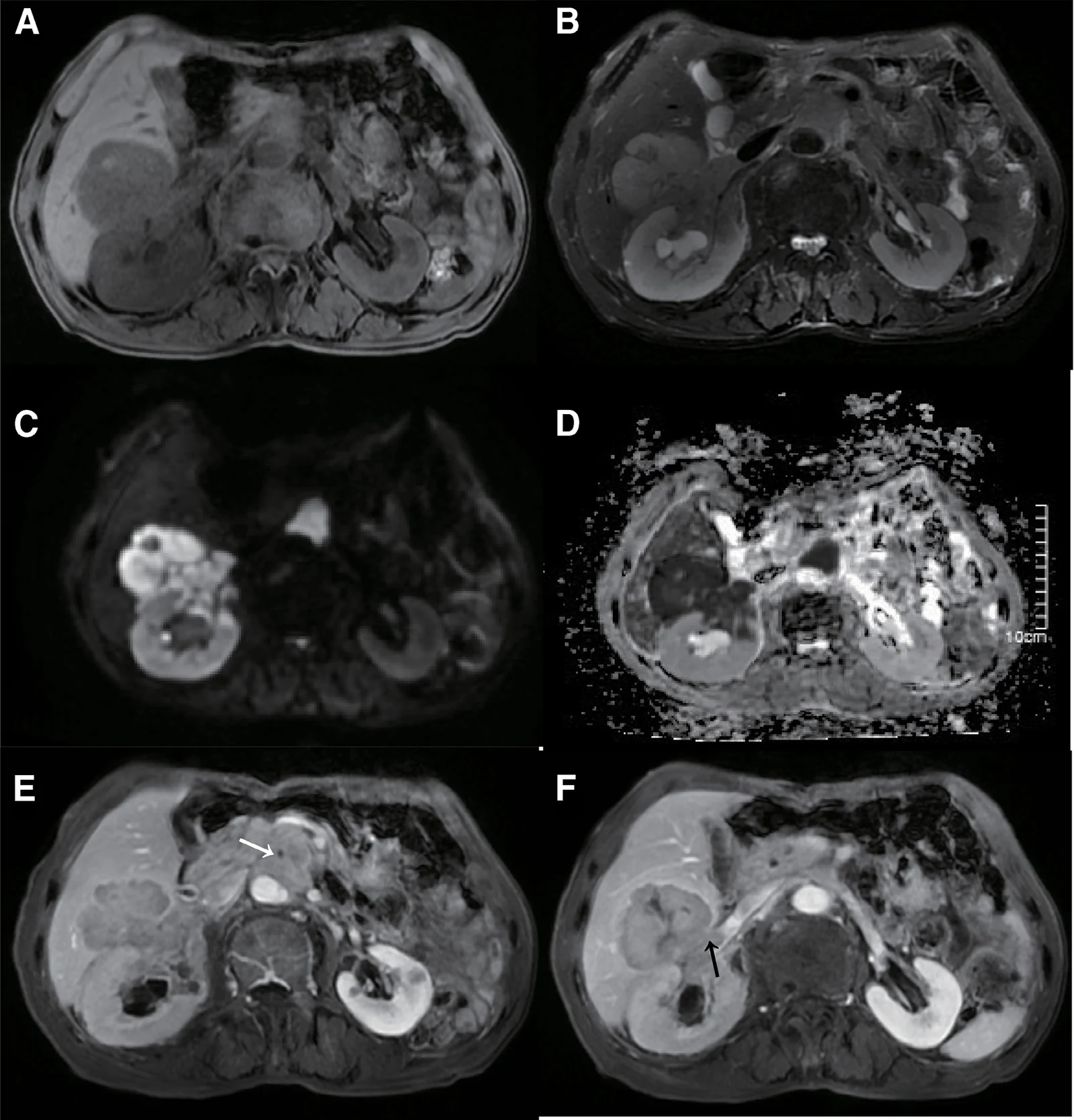
Contrast-enhanced MRI (CE-MRI). (A) The mass shows low signal on T1-weighted imaging (T1WI). (B) The mass shows high signal on T2-weighted imaging (T2WI). (C) The mass demonstrates high signal on diffusion-weighted imaging (DWI). (D) The apparent diffusion coefficient (ADC) map shows markedly reduced signal, indicating restricted diffusion. (E) CE-MRI demonstrates uniform moderate enhancement of the mass. (F) The mass infiltrates the renal vessels (black arrow). The white arrow indicates a para-aortic lymph node.

Contrast-enhanced ultrasonography (CEUS) was performed on the mass using a Mindray Resona R9T system with a C5–1 transducer (frequency range: 1–5 MHz). SonoVue was administered at a dose of 1.0 mL via manual bolus injection, followed by a 5 mL saline flush through a peripheral venous line in the left forearm. CEUS assessment was conducted by a physician with 3 years of CEUS experience. CEUS (Fig. 4) showed rapid contrast enhancement with central necrosis and arterial isoechoic enhancement, followed by hypoechoic enhancement during the parenchymal phase. Although central necrosis and an expansile growth pattern can be seen in RCC, the CEUS findings in this case were interpreted as supporting the initial diagnosis of RCC. Under the initial suspicion of RCC, an ultrasound-guided renal needle biopsy (Fig. 5) was performed. Histopathology revealed lymphoid cell infiltration with marked crush artifact in fibrocollagenous tissue, consistent with DLBCL. Immunohistochemically, the neoplastic large B-cells were diffusely positive for CD20 and CD79a, confirming B-cell lineage. The tumor cells also expressed Bcl-2 (90%+), Bcl-6 (+), and MUM1 (70%+), and showed a high proliferative index (Ki-67 approximately 50%); based on the Hans algorithm, this immunophenotype is consistent with the non-GCB subtype of DLBCL. Subsequently, a positron emission tomography–computed tomography (PET-CT) scan (Fig. 6) was performed. FDG-PET revealed no other foci of uptake besides the retroperitoneal lymph nodes.

**Figure 4.**
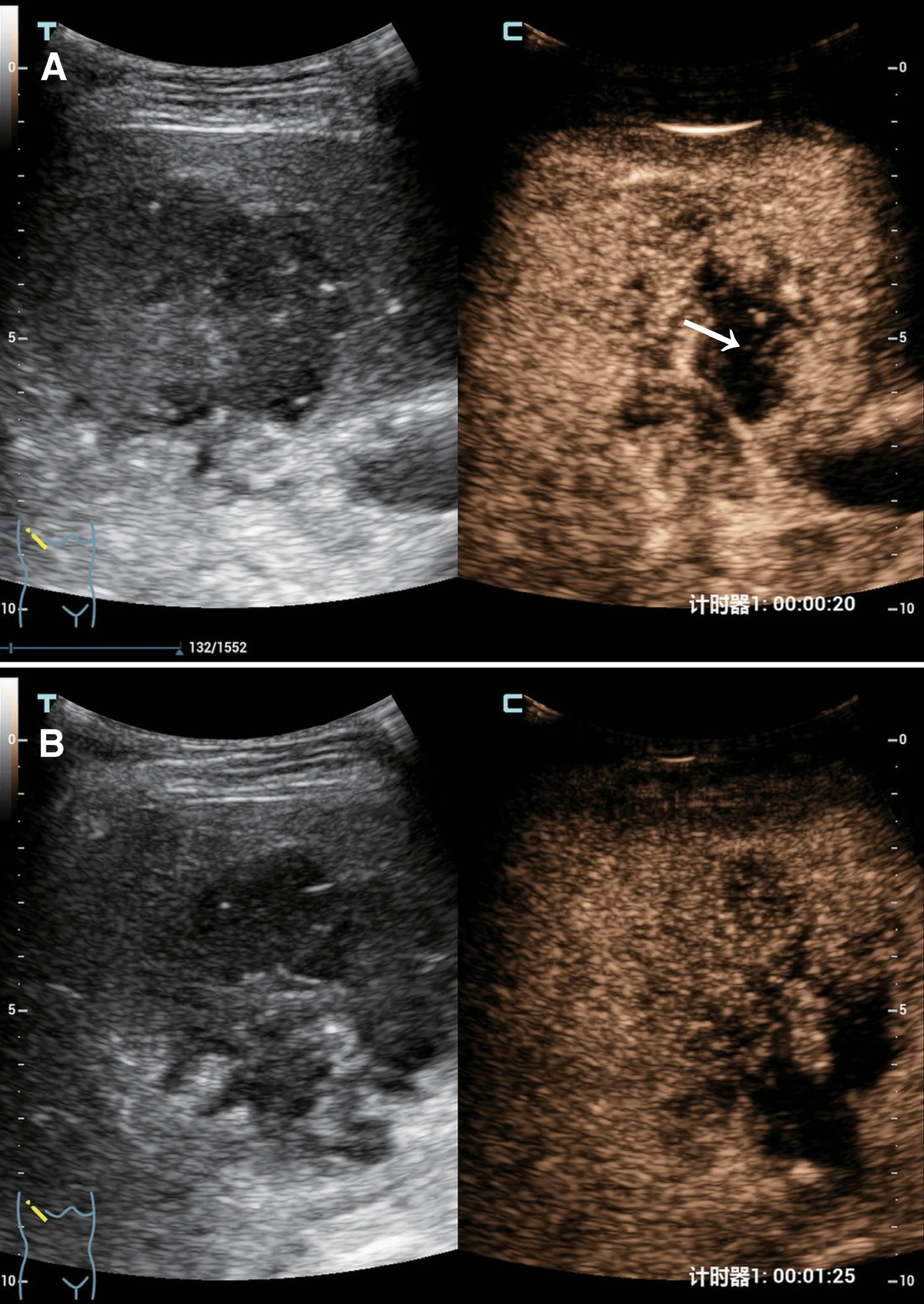
Contrast-enhanced ultrasonography (CEUS). (A) In the arterial phase (20s post injection), the mass contains necrotic areas. (B) In the venous phase (85s post injection), the mass appears hypoenhanced. The white arrow indicates necrotic areas.

**Figure 5.**
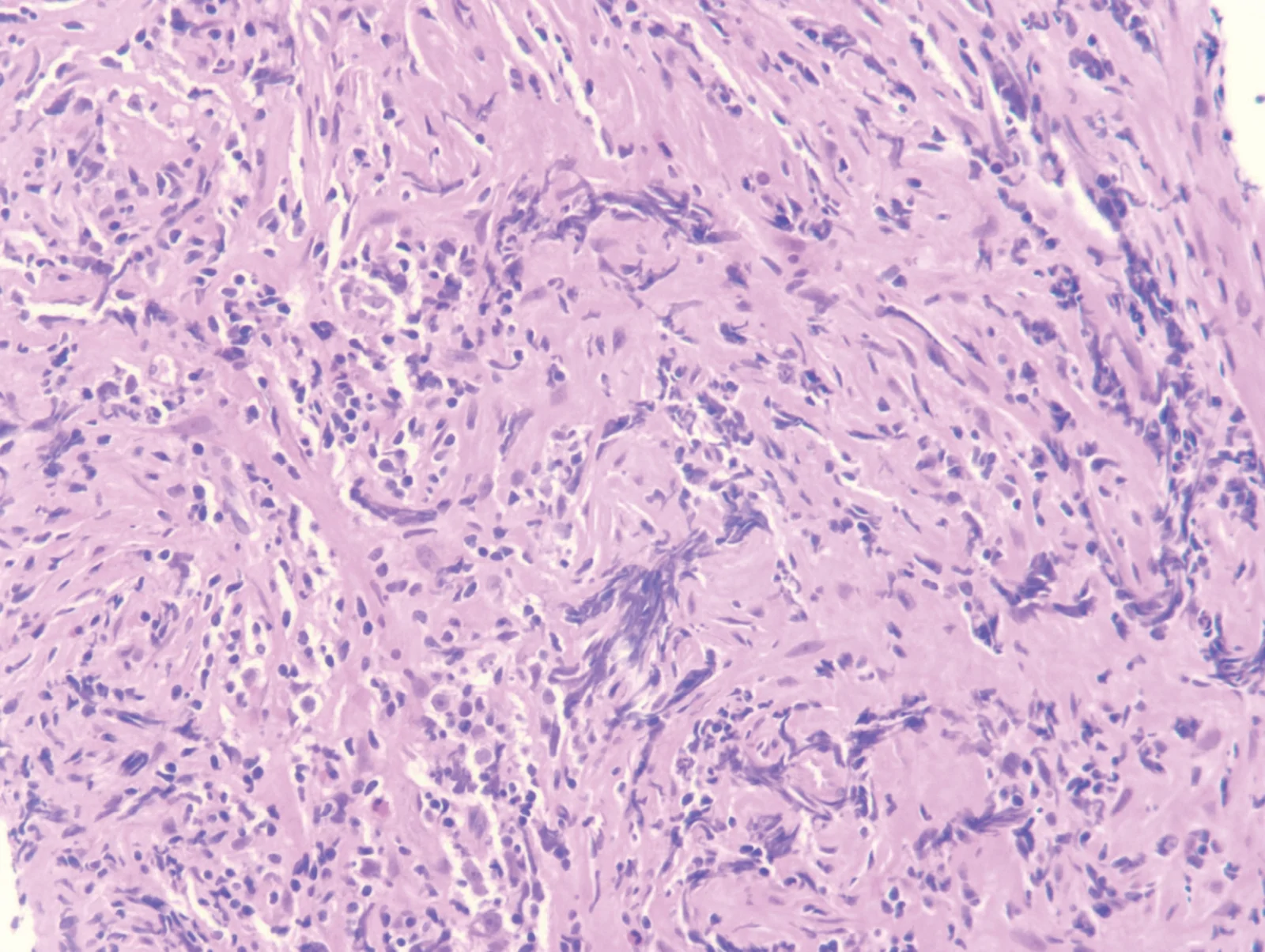
Histology. Biopsy showing diffuse large B-cell non-Hodgkin lymphoma involving the right kidney (H&E stain, magnification × 100). H&E = hematoxylin and eosin stain.

**Figure 6.**
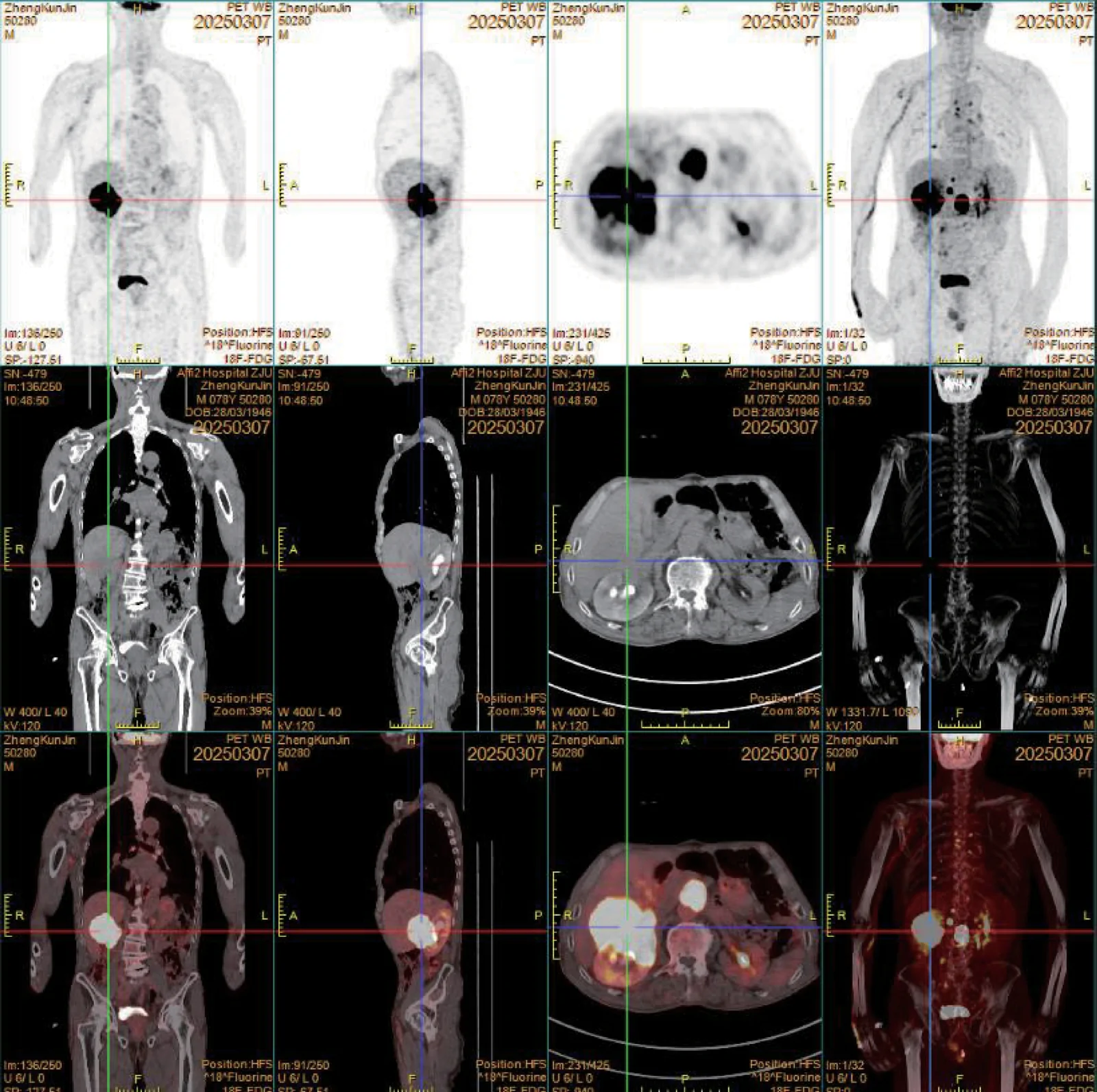
PET-CT. No other foci of uptake besides the retroperitoneal lymph nodes were detected on PET-CT. PET-CT = positron emission tomography computed tomography.

Regrettably, the patient declined treatment, and follow-up information is unavailable.

## 3. Discussion

Lymphoma refers to malignancies of the lymphoid tissues and is broadly classified into Hodgkin lymphoma and NHL.^[5]^ While kidney involvement is common in advanced NHL, PRL is rare.^[5,6]^ PRL is an aggressive neoplasm with distinct pathological and clinical features and is estimated to account for <1% of all renal malignancies. No universally accepted diagnostic criteria for PRL exist. Most published criteria require – a renal mass pathologically confirmed as lymphoma; no lymph node or visceral organ involvement outside the kidney – although limited (small-volume) retroperitoneal nodal involvement does not necessarily exclude the diagnosis;^[7,8]^ no leukemic blood picture or bone marrow suppression; and no emergence of lymphoma at other sites within 3 months of the initial diagnosis.^[7]^ In our case, the tumor originated in the right kidney with no evident lesions elsewhere, fulfilling the criteria for PRL.

Although PRL commonly presents with fever, fatigue, anorexia, night sweats, flank discomfort, or an abdominal mass, these symptoms are nonspecific.^[9]^ As such, PRL is frequently confused with RCC, as in the index case.^[10–12]^ Patients with PRL who present with typical RCC symptoms such as gross hematuria, low back pain, abdominal mass, or renal dysfunction are often misdiagnosed as having RCC, given its greater prevalence. Despite the symptom overlap in our patient, PRL was not initially included in the differential diagnosis due to this common imaging overlap.

Diagnosing PRL requires the integration of clinical, laboratory, multiple imaging, and ancillary studies. Ultrasonography, CEUS, CT, MRI, PET-CT, bone marrow examination, immunohistochemistry, and cytogenetics all play important roles in PRL evaluation. Ultrasonography is often the first imaging modality in patients with renal symptoms and can help characterize lesions and distinguish solid tumors from pseudo-lesions and complex cysts;^[13]^ however, it is usually insufficient to differentiate renal lymphomas from other malignant renal masses.^[14]^

CEUS, which is safe in patients with impaired renal function,^[15]^ can depict the enhancement patterns of renal masses during the arterial and parenchymal phases and use the surrounding renal parenchyma as an in vivo reference. Typically, PRL shows uneven or weak enhancement and usually lacks central necrosis, whereas RCC often demonstrates persistent early enhancement, with central necrosis being common.^[16,17]^ Notably, our case – though ultimately diagnosed as DLBCL, a common PRL subtype – exhibited central necrosis on CEUS, an atypical feature for PRL. Two possible explanations may account for the atypical manifestations observed in this case. First, CEUS contrast agents are intravascular agents that enhance the visibility of the microvasculature and reflect tissue perfusion. In the present case, the rapid growth of the tumor may have outpaced its blood supply, leading to ischemia and subsequent central necrosis. Second, the densely packed and highly invasive nature of the tumor cells may have compressed the central vasculature, thereby reducing or abolishing blood flow within the central area of the lesion. These hypotheses are speculative and warrant further investigation.

CT remains the imaging modality of choice for initial staging, but renal lymphoma can show limited enhancement due to relatively poor vascularity, complicating differentiation from RCC. MRI can better delineate renal and perirenal involvement, with PRL typically showing lower enhancement than normal renal parenchyma and possible progressive enhancement on delayed imaging.^[18]^ Renal vascular invasion and tumor thrombus formation are uncommon in PRL but frequently encountered in RCC.^[17,18]^ Our case suggests that renal lymphoma may also exhibit renal vascular invasion; however, this represents only an initial observation derived from a single case and warrants further validation in larger cohorts. PET-CT is valuable for staging lymphoma and detecting metabolically active disease.^[19]^ It is generally more sensitive than conventional anatomical imaging for identifying additional foci and for assessing treatment response and surveillance. In this case, FDG-PET revealed no abnormal uptake outside the kidneys except in the retroperitoneal lymph nodes. Importantly, limited retroperitoneal nodal involvement does not exclude the diagnosis of PRL, as the retroperitoneum constitutes the locoregional drainage territory of the kidney.^[7,8]^ In the present case, only a few small retroperitoneal lymph nodes (<4 cm) were detected on FDG-PET, with no other extrarenal FDG-avid lesions, fulfilling the criteria for limited locoregional nodal involvement rather than systemic disease. Therefore, these findings are consistent with the diagnosis of PRL, and the observed retroperitoneal nodes are regarded as regional lymphatic spread from the primary renal lesion.

Notably, our case underscores the clinical value of renal biopsy. Primary DLBCL is a rare entity that can be challenging to differentiate from other renal masses. Renal biopsy is critical for differential diagnosis and enables definitive confirmation of PRL, especially in patients without a prior history of lymphoma.^[10]^ Ultrasound-guided renal core needle biopsy provides real-time imaging guidance and avoids radiation exposure. CEUS may further improve needle visualization and help distinguish viable solid tumor tissue from necrotic areas.

The prognosis of PRL is difficult to determine because of its rarity; however, available data suggest unfavorable survival, with a 5-year overall survival rate not exceeding 40–50%.^[7]^ Outcomes appear to have improved since the introduction of rituximab, with population-based data reporting a 5-year relative survival of approximately 64%.^[20]^ Unlike RCC, which is primarily managed surgically, PRL is a systemic disease, and chemo immunotherapy is the cornerstone of its treatment.^[7]^ No clinical trials or formal guidelines specific to PRL exist, and treatment is therefore based on regimens established for nodal DLBCL. The most commonly used regimen is R-CHOP (rituximab, cyclophosphamide, doxorubicin, vincristine, and prednisone), administered for 6 to 8 cycles; most patients achieve a complete or partial response.^[21]^ Therefore, differentiating PRL from RCC before treatment is crucial, as a biopsy-based preoperative diagnosis can prevent unnecessary nephrectomy and allow prompt initiation of chemo immunotherapy.

A limitation of this case report is that treatment response and outcome could not be assessed owing to the patient’s refusal of treatment and follow-up evaluation.

In conclusion, PRL represents a rare, aggressive malignancy with an insidious onset and an unfavorable prognosis. It can radiologically mimic multiple other malignant renal lesions, posing substantial diagnostic difficulties. Our case emphasizes that PRL should be included in the differential diagnosis for large renal space-occupying lesions in clinical practice. Moreover, it highlights the essential role of ultrasound‑guided renal needle biopsy for renal masses, rather than relying solely on imaging modalities.

## Acknowledgments

The authors thank all the participants of the Department of Ultrasound, The Second Affiliated Hospital, School of Medicine, Zhejiang University, for their valuable contributions. The authors thank the selfless sharing by the patients. The authors thank Editage (www.editage.cn) for English language editing.

## Author contributions

**Conceptualization:** Xintong Li, Shijie Sun.

**Data curation:** Xintong Li, Shijie Sun.

**Writing – original draft:** Xintong Li.

**Writing – review & editing:** Jia Li, Pintong Huang.
